# Video analysis of cardiopulmonary resuscitation performance of ambulance crews during transportation

**DOI:** 10.1186/cc13673

**Published:** 2014-03-17

**Authors:** H Giga, T Otani, T Sadamori, K Une, Y Kida, K Ota, J Itai, S Yamaga, S Kusunoki, S Ohshimo, Y Iwasaki, N Hirohashi, K Tanigawa

**Affiliations:** 1 Hiroshima University, Hiroshima, Japan; 2 Critical Care Medical Center, Hiroshima Prefectural Hospital, Hiroshima, Japan

## Introduction

High quality of chest compressions during cardiopulmonary resuscitation (CPR) is a critical determinant of outcome from out-of-hospital cardiac arrest (OHCA). Unfortunately, however, victims often do not receive adequate chest compression for various reasons, particularly during transportation. Recent studies have demonstrated the interruption time of chest compression using transthoracic impedance analysis, but more information is needed to evaluate the performance of CPR provided by ambulance crews and reveal reasons for hands off chest during CPR.

## Methods

All ambulances of the Hiroshima City Fire Department are equipped with a specially designed transmission device (RVT-SD200; Sony) that transmits high-resolution visual images and patient vital data using video cameras and a bio-monitor. We analyzed video data of OHCA patients transported by ambulance from November 2012 through December 2012, and evaluated the performance of CPR during transportation in accordance with the 2010 guidelines. The hands-off time was calculated as the time without chest compressions divided by the total CPR time.

## Results

Thirty-two resuscitation episodes during transportation by ambulance were analyzed. Median CPR time per episode was 846 seconds (range 126 to 1,833 seconds). In total, the fraction of time without chest compression was 19.5 ± 7.6% (mean ± SD). Reasons for interruption and its fraction of time in total hands-off time were as follows: 36% accounted for rhythm analysis/pulse check, 31% for ventilation, 11% for setting up automated chest compression devices, 8% for tracheal intubation/placement of supraglottic airway devices, 4% for intravenous line placement/administration of adrenaline, 3% for rescuer change, and 7% for adjustment of patient position/correction of rescuer posture and others.

## Conclusion

The fraction of time without chest compression observed in this study was comparative with those found in other studies in spite of the difficult situations, such as during transportation. Most frequent reasons for hands-off time were rhythm analysis and ventilation even though the ambulance crews strictly adhered to the guidelines.

